# “Inside Out”: A Regional Inpatient Joy in Work Project

**DOI:** 10.1192/bjo.2022.319

**Published:** 2022-06-20

**Authors:** Tammy Morgan, Claire Kelly

**Affiliations:** Beechcroft, Belfast, United Kingdom

## Abstract

**Aims:**

Inpatient admissions during the COVID-19 pandemic went up in the regional unit by 18%. This included a 50% increase in Eating Disorder Presentations and more complex SMI requiring admission to Beechcroft. Beechcroft is the regional inpatient unit for CAMHS in Northern Ireland. This project aimed to improve staff joy in work by 30% by June 2021, following what was one of the most difficult years to be a health professional with the COVID-19 pandemic.

**Methods:**

We used the IHI Joy in Work methodology along with our own rating scales in the inpatient unit.

Several PDSA cycles were carried out including focus groups, gathering baseline data from different wards, and our change ideas- Beechcroft stars nominations, Virtual Quizzes and Staff recognition certificates.

**Results:**

Baseline data on our run chart demonstrated a bad day median in Beechcroft with 4.3 being the score.

With the PSDA cycle we demonstrated a 33% improvement in good day scores with a median of 1.4.

We have learnt that Joy in work comes from recognising the work already being done and rewarding the efforts our staff go to.

Spread and scale with Beechcroft stars now part of fortnightly MDT meeting and management meeting. Also rolled out to a community mental health team.

**Conclusion:**

Joy in Work comes from the team. Recognising the efforts of the team is central to this. In particular during a pandemic.

